# Optical Coherence Tomography in Patients with Chiari I Malformation

**DOI:** 10.1155/2015/756261

**Published:** 2015-02-22

**Authors:** Michele Figus, Chiara Posarelli, Francesco Nasini, Paolo Perrini, Mario Miccoli, Angelo Baggiani, Antonio Ferreras, Marco Nardi

**Affiliations:** ^1^Department of Surgical, Medical, Molecular Pathology and Emergency, University of Pisa, 56124 Pisa, Italy; ^2^Department of Neurosurgery 2, University of Pisa, 56124 Pisa, Italy; ^3^Department of Translational Research and Advanced Technologies in Medicine and Surgery, University of Pisa, 56124 Pisa, Italy; ^4^Miguel Servet University Hospital, Aragon Health Sciences Institute, Zaragoza, Spain

## Abstract

*Background/Aims*. To evaluate optic nerve head with spectral domain optical coherence tomography (OCT) in patients with Chiari I malformation (CMI) compared to healthy controls. *Methods*. Cross-sectional study. OCT of the optic nerve head of 22 patients with CMI and 22 healthy controls was quantitatively analyzed. The healthy controls were matched for age and sex with the study population. Mean retinal nerve fiber layer (RNFL) thickness was calculated for both eyes; the mean thickness value was also registered for each quadrant and for each subfield of the four quadrants. *Results*. CMI patients showed a reduction of the RNFL thickness in both eyes. This reduction was more statistically significant (*P* < 0.05) for the inferior quadrant in the right eye and in each quadrant than nasal one in the left eye. *Conclusion*. A distress of the retinal nerve fibers could explain the observed reduction of the RNFL thickness in patients with CMI; in our series the reduction of the RNFL thickness seems lower when CMI is associated with syringomyelia.

## 1. Introduction

Chiari I malformation (CMI) is a rare congenital disorder recognized by caudal displacement of the cerebellar tonsils through the foramen magnum and into the cervical canal [1]. The mechanism for the development of the often associated syringomyelia is attributed to partial obstruction of the subarachnoid space at the foramen magnum due to the pulsatile impaction of the tonsils [2, 3]. The complex symptom patterns for CMI usually are late-onset and are related to the presence of an associated syrinx and/or the compression of neural structures at the cervicomedullary junction by the herniated tonsils [4]. The most common presenting symptom is suboccipital headache, which has been reported in 81% of patients and is classically exacerbated by Valsalva maneuvers [5]. Additional presenting symptoms include ocular, otoneurological, brainstem, and spinal cord disturbances [5]. Symptoms involving ocular function consist of pseudotumor-like episodes of blurred vision, photophobia, diplopia, retroorbital pain, and visual fields cuts and occur in 78% of patients [5]. Despite the high percentage of this association, the neuroophthalmological findings of patients with CMI have not been extensively investigated in literature and remain largely elusive. Looking at the ophthalmological examination papilloedema is a rare but known manifestation of CMI [5–10]. This study aimed to observe the RNFL thickness through optical coherence tomography (OCT) in a consecutive series of patients with CMI compared with a control group.

## 2. Materials and Methods

Twenty-two patients who had been referred to neurosurgery department for evaluation of CMI were investigated between March 2011 and July 2012. Patients with any coexisting ocular disease were excluded, but otherwise there was no patient selection.

Healthy controls were recruited from staff at the hospital; people with known ophthalmologic or other neurologic diseases were excluded.

The work was conducted in accordance with the Declaration of Helsinki (1964) and the consents of the human subjects were collected. Any ethical committee authorization was necessary because of observational study. Complete clinical and neuroradiological assessment was performed in all patients. Neuroradiological investigation included magnetic resonance (MR) imaging of the cranial and spinal compartments. To be eligible for the study, each patient was required to have cerebellar tonsillar ectopia measured as ≥5 mm below a line connecting the basion and the opisthion on MR images. Tonsillar ectopia was quantified by measuring a line perpendicular to this line that extended to the most inferior aspect of the cerebellar tonsils [11–13]. Additionally, MR images were assessed for the presence of syringomyelia, a chronic disease of the spinal cord characterized by the presence of fluid-filled cavities and leading to spasticity and sensory disturbances.

Syringomyelia is a dilatation caused by excessive cerebrospinal fluid (CSF) accumulation within the central canal or the extracanalicular area. Type 1 and 2 Chiari, the Dandy-Walker syndrome, cerebellar ectopia, basilar impression, basilar arachnoiditis, posterior fossa tumors and arachnoid cysts, medulla spinalis tumors, spinal trauma, and infections are among the pathologies that can cause syringomyelia. There are also several idiopathic cases of syringomyelia.

Our study cohort consists of 22 patients and 22 healthy controls. The healthy controls were matched for age and sex with the study population and to be eligible for the study the clinical, neuroradiological, and ophthalmological examination should be negative for any known disease.

All the study population underwent clinical examination which included ophthalmological evaluation, which consisted of best corrected visual acuity (BCVA) and intraocular pressure (IOP) measurement and fundus examination. BCVA was registered using logarithm of the minimum angle of resolution (LogMAR).

**Figure 1:**
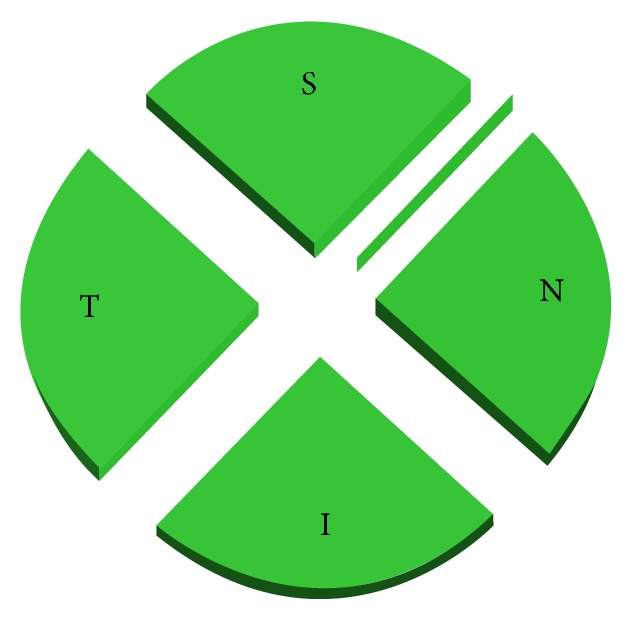
The peripapillary area has been divided in four quadrants.

**Figure 2:**
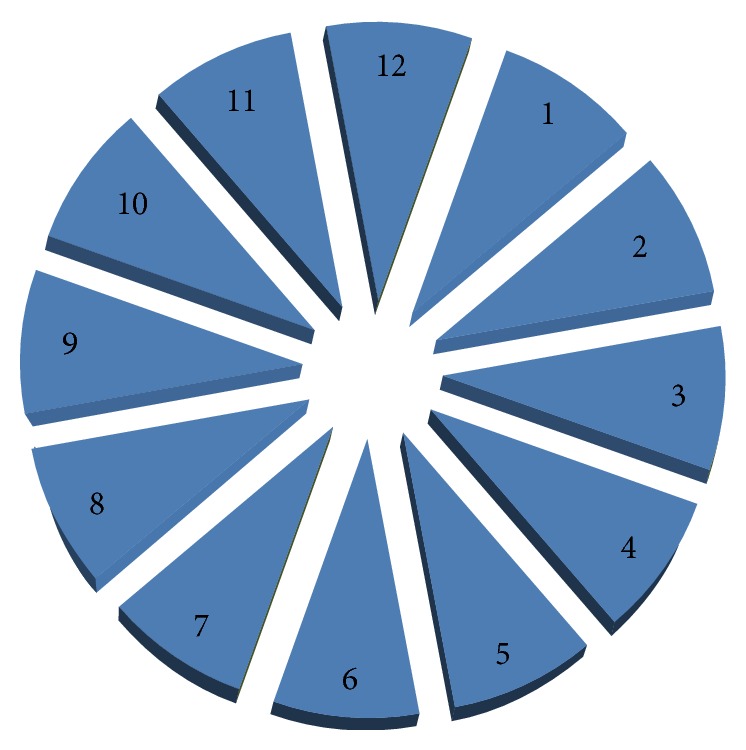
The peripapillary area has been divided in 12 subfields, three for each quadrant.

The Topcon 3D OCT 1000 (Topcon, Oakland, NJ, version 3.30) uses a superluminescent diode laser with a wavelength of 840 nm as the light source. This system acquires 27,000 A-scans per second with axial resolution of ≤6 *μ*m. The protocol used was the Glaucoma 3D 6.6 mm, which includes 128 horizontal scans comprised of 512 A-scans. Only scans with quality factor (Q-factor) ≥45 were accepted. After creating a peripapillary RNFL thickness map from the cube dataset in both devices, the software finds the optic disc and automatically places a calculation circle 3.46 mm in diameter centered around the disc; the machine then extracts the peripapillary circle data from the cube dataset for RNFL thickness measurement. Internal fixation was used for all subjects with both devices and if fixation was not proper, an external fixation target was applied in order to move the scanning area to the appropriate location. Images with discontinuity or misalignment, involuntary saccade, or blinking artifacts were excluded. The RNFL thickness parameters that were automatically calculated by the machines and investigated in this study included average thickness, temporal, superior, nasal, and inferior quadrant thicknesses, and three subfields for each quadrant thicknesses (Figures 1 and 2).

Among the 22 patients, 19 patients with mild or absent clinical symptoms were not considered for immediate surgical treatment, whereas 3 symptomatic patients underwent posterior fossa decompression, dura mater opening, and duraplasty. In the surgical group, the first postoperative clinical and neuroradiological examinations (MR) were performed 3 months after surgery.

Statistical analysis was performed using the Shapiro-Wilk test to verify normality of distributions in order to assess whether or not to use parametric tests. We used the* t*-test to compare Gaussian distributions; otherwise we performed the Mann-Whitney* U* test. The statistical analysis was concluded with power tests (ex-post) to estimate the sample sizes required for the study. The 1−*β* value of the significant variables was >0.8, assuring a low risk of type II error and an appropriate sample size. Data were entered into an Excel database and were subsequently imported into the software IBM SPSS Statistics 17.0.1.

## 3. Results

Twenty-two patients affected by CMI, ranging in age from 17 to 54 years (mean age, 35 years), were enrolled; 15 patients were women and 7 were men (3 : 1). Mean BCVA was 0.0 LogMAR in both eyes, and mean IOP was normal, respectively, 13.6 ± 2.6 mmHg in right eye (RE) and 13.9 ± 2.6 mmHg in left eye (LE). At the fundus examination the ONH appearance was normal in all patients except for 3 symptomatic women that showed a papilloedema. In six of the twenty-two patients at the magnetic resonance (MR) imaging of the cranial and spinal compartments a syringomyelia was detected; for this reason the patients were divided into two groups, the first one with CMI alone (Group A, sixteen patients) and the second group with CMI associated with syringomyelia (Group B, six patients). None of the patients complained from diplopia, and only two had blurry vision.

Twenty-two healthy individuals were included in the study as controls. The mean age was 38 years (min 25; max 52). BCVA was 0.0 LogMAR in all cases, and mean IOP was, respectively, 14.2 ± 1.5 mmHg in RE and 15.5 ± 2.1 mmHg in LE (normal values < 21 mmHg) comparable to the group of patients. Fundus examination and OCT images were unremarkable. As for the case group we divided the controls into two groups (Group C and Group D) matched for age and sex to the case groups. For each patient we considered the OCT examination of the right eye and of the left one and we compared it, respectively, with the right eye and the left eye of the controls.

The mean RNFL thickness in CMI patients (Group A) was more inferior than in control patients (Group C) with a statistically significant reduction (*P* < 0.05) in the RE in the inferior quadrant and in a subfield of the temporal quadrant; in the left eye the reduction of the mean RNFL thickness was significant and also in three of the four quadrants (superior, temporal, and inferior) the reduction was statistically significant (Tables 1 and 2).

Comparing Group B (CMI associated with syringomyelia) to the control group (Group D) a smaller reduction of the mean RNFL was observed but the difference was statistically significant (*P* < 0.05) in the superior quadrant of the RE and in the temporal quadrant of the LE; a statistically significant (*P* < 0.05) difference was also observed in four subfields: one in the RE and three in the LE (Tables 3 and 4).

From the comparison between patients with CMI (Group A) and patients with CMI and syringomyelia (Group B) a statistically significant (*P* < 0.05) difference has been observed for the mean RNFL thickness of the RE and in the inferior quadrant of the LE, showing a higher value in Group B.

The three patients that underwent surgery were observed before the surgical procedure and three months later, one of them had CMI alone and the other two had CMI and syringomyelia; any significant reduction in RNFL thickness was observed.

## 4. Discussion

OCT images of the optic nerve head and analysis of the mean RNFL thickness associated with the mean thickness value of the four quadrants allowed detailed quantitative description of the peripapillary RNFL. We observed a reduction of the mean RNFL thickness in CMI patients when compared to healthy controls, but this reduction is lower if we consider CMI patients with syringomyelia and surgical patients with CMI. Probably it could be related to the papilloedema more frequently observed in patients with syringomyelia and surgical patients with CMI, as previously documented in literature [6, 7]. This is an explorative study; for this reason we decided to consider separately the two eyes and not to randomize only one eye. Looking at the results LE seems more severely affected than the right one, but CMI is a central condition and we expected to find any difference between the eyes. Looking at the published literature [7–10] a different involvement of the eyes is described; particularly papilloedema seems greater in one eye than in the other. Further larger studies are necessary to confirm if there could be a difference between the eyes and if this difference is significant when compared with the controls.

Zhang et al. [6] described in their paper the case of a woman with symptomatic CMI and papilloedema, OCT documented, and treated surgically with suboccipital decompression. After surgery the patient's visual symptoms improved but a residual bilateral optic nerve atrophy was observed. In fact, it is crucial to differentiate CMI and idiopathic intracranial hypertension, because of the similarities in patient's presentation [6, 14]. Bilateral optic disc oedema with preserved visual acuity and retinal nerve fiber layer loss is typically the consequence of papilloedema. And this could explain the reduction of RNFL observed in our patients.

The pathogenesis of papilloedema in CMI is not clear. Some authors [2, 7] speculated that posterior fossa crowding results in cerebrospinal fluid flow delay, causing spikes of intracranial pressure (ICP) during pressure pulses (Valsalva maneuvers, etc.). On the other side it is likely that there exist some structural congenital changes that predispose to development of papilloedema. This could explain why not all CMI patients, independently from the cerebellar morphology, have a papilloedema. It is likely that even if the edema is not visible the chronic tonsilar herniation determines nerves fiber suffering that justifies a thinner RNFL in CMI patient than healthy subjects. But this could also explain why this difference is less when syrinx is associated with CMI. This group of patients showed a thicker optic nerve head probably due to an oedema of the ONH.

Vaphiades et al. [7] described a case series of four patients with CMI successfully treated with suboccipital decompression. The authors stated that the primary role of neuroimaging in patients with papilloedema is to exclude hydrocephalus and disorders that cause increased ICP, particularly space-occupying lesions and cerebral venous thromboses. They also concluded that in their experience posterior fossa decompression can be curative in patients with increased ICP and papilloedema caused by CMI. In our series only three patients underwent suboccipital decompression. All of them showed a papilloedema but looking at the RNFL thickness three months after surgery any significant reduction was observed. Probably the three-month ophthalmological evaluation was too early to identify a modification. Two of the patients presented CMI associated with syringomyelia, so in Group B two of the 6 patients underwent decompression and this could represent a statistical bias, but we decide not to exclude them from the comparison with healthy controls because this is an explorative study and we try to obtain as much as we can from the analysis.

Recent OCT studies in neuroophthalmological diseases [15–17] like multiple sclerosis (MS) or Parkinson's disease showed a reduction of the RNFL thickness; Pulicken et al. [15] observe that RNFL is significantly decreased in multiple sclerosis optic neuritis (ON) eyes, unaffected fellow eyes of patients with MS ON, and MS eyes not affected by ON. This has suggested that chronic axon damage can occur in MS eyes distinct from acute ON episodes. They also observed that subclinical ON could also damage axons in progressive forms of MS. These findings could be explained either by a subclinical ongoing inflammation or by neuronal cell death. CMI as MS is a chronic condition that could lead to neuronal cell death and axons damage.

Schild et al. [16] described a reduction of the RNFL thickness as well as ganglion cell layer (GCL) in patients with autosomal dominant optic atrophy using spectral domain OCT (SDOCT) images, especially in the inferior and superior peripapillary quadrants. The authors concluded that SDOCT volume scans could be important to diagnose a loss of vision of unclear origin but also to see the effects of future therapies. Equally in CMI patients, OCT could represent an examination to quantify the ONH oedema and to follow the patients after surgery besides neuroimaging. Equally Feng et al. [17] demonstrated in Chinese people with MS the thinning of RNFL with SDOCT and they suggested the use of the tomography as a high-resolution, objective, noninvasive, and easily quantifiable in vivo biomarker of MS. OCT is a rapid high-resolution method to obtain morphological and quantitative information that could be important in the management and follow-up of patients with neurodegenerative diseases such as CMI or SM. At least looking at the studies of Garcia-Martin and her research group in Saragoza [18, 19], we obtain a confirmation of what we have already discussed for MS, because the analysis based on the segmentation technologies of the Spectralis OCT (Heidelberg Engineering, Inc., Heidelberg, Germany) showed retinal layer atrophy in patients with MS, especially of the inner layers. They observed also the reduction of the ganglion cell and inner plexiform layer as predictor of greater axonal damage. The Spanish group study [19] observed also with the same technique Parkinson's disease and SDOCT revealed retinal layer atrophy especially in patients with longer duration disease.

One limitation of this study is that we consider a small study group, but CMI is a rare disease. Although this is unlikely to affect the results, it may introduce a possible bias in the study. In addition, it is possible that some individuals with low-tension glaucoma were included in both groups (CMI and controls), which may have biased the findings. Considering the not statistically significant difference between pre- and postsuboccipital decompression, the short time (only three months) follow-up could explain our results. Further studies are needed to confirm our findings and to determine whether there could be a difference between the four quadrants of the optic nerve head or if there could be a difference between eyes.

In conclusion OCT of the optic nerve head in CMI patients represents a reproducible, noninvasive, high speed, and high-resolution instrument that can add useful information for the treatment of these patients. OCT measurements are tools that can be used in combination with other parameters and clinical explorations such as neuroimages.

This technique may further be important also for the documentation of possible future therapies and for the follow-up of nonsurgical patients to monitor the chronic fiber suffering and axon cell death through the thinning of the RNFL in a long-term follow-up time.

## Figures and Tables

**Table 1:** CMI patients (Group A) versus healthy subject (Group C) OCT parameters, RE.

Variables	Average (*µ*)	Average (*µ*)	*P* value
	Standard deviation Group A	Standard deviation Group C	
RNFL	102,750 (7,188)	108,500 (14,180)	0.158
Superior quadrant	130,813 (17,387)	127,688 (17,327)	0.614
Subfield 11	124,375 (20,314)	133,313 (20,959)	0.230
Subfield 12	139,000 (25,451)	129,813 (26,838)	0.328
Subfield 1	129,063 (23,809)	119,875 (26,339)	0.309
Nasal quadrant	94,000 (17,877)	96,500 (23,653)	0.738
Subfield 2	124,375 (20,314)	133,313 (20,959)	0.534
Subfield 3	139,000 (25,451)	129,813 (26,838)	0.759
Subfield 4	129,063 (23,809)	119,875 (26,339)	0.902
Inferior quadrant	115,438 (14,882)	129,688 (17,861)	0.020^*^
Subfield 5	104,750 (17,935)	110,563 (32,307)	0.115
Subfield 6	80,313 (20,921)	82,500 (19,082)	0.142
Subfield 7	96,625 (21,713)	95,625 (23,872)	0.068
Temporal quadrant	94,000 (17,877)	96,500 (23,653)	0.454
Subfield 8	103,250 (27,560)	118,500 (25,612)	0.262
Subfield 9	121,688 (25,933)	135,750 (26,792)	0.118
Subfield 10	122,000 (16,959)	134,250 (19,608)	0.035^*^

RNFL: retinal nerve fiber layer thickness in *μ*.

Group A: patient with CMI.

Group C: healthy subjects.

1–12: three subfields for each quadrant numbered clockwise.

^*^ *P* < 0.05, *t*-test.

**Table 2:** CMI patients (Group A) versus healthy subject (Group C) OCT parameters, LE.

Variables	Average (*µ*)	Average (*µ*)	*P* value
	Standard deviation Group A	Standard deviation Group C	
RNFL	98,563 (10,09)	98,563 (10,099)	0.007^*^
Superior quadrant	119,688 (16,756)	127,438 (15,900)	0.035^**^
Subfield 11	121,500 (38,346)	131,375 (26,051)	0.800
Subfield 12	125,688 (23,431)	124,125 (19,280)	0.838
Subfield 1	107,125 (16,721)	126,813 (25,079)	0.014^*^
Temporal quadrant	65,813 (10,381)	85,563 (10,381)	0.0003^*^
Subfield 2	74,375 (11,960)	95,875 (16,358)	0.0002^*^
Subfield 3	52,750 (9,356)	74,375 (18,319)	0.0002^*^
Subfield 4	70,188 (13,905)	85,500 (19,375)	0.017^*^
Inferior quadrant	118,813 (14,616)	134,938 (20,780)	0.017^*^
Subfield 5	131,438 (22,091)	142,313 (28,109)	0.233
Subfield 6	124,063 (26,839)	147,188 (35,309)	0.046^*^
Subfield 7	101,625 (18,446)	115,875 (36,138)	0.170
Nasal quadrant	89,250 (25,104)	93,188 (21,097)	0.454
Subfield 8	79,688 (25,742)	88,750 (18,109)	0.259
Subfield 9	75,625 (22,295)	80,250 (19,797)	0.540
Subfield 10	111,563 (32,356)	104,250 (39,079)	0.569

RNFL: retinal nerve fiber layer thickness in *μ*.

Group A: patient with CMI.

Group C: healthy subjects.

1–12: three subfields for each quadrant numbered clockwise.

^*^ *P* < 0.05, *t*-test.

^**^ *P* < 0.05, Mann-Whitney *U* test.

**Table 3:** CMI patients with syringomyelia (Group B) versus healthy subject (Group D) OCT parameters, RE.

Variables	Average (*µ*)	Average (*µ*)	*P* value
	Standard deviation Group A	Standard deviation Group C	
RNFL	112,667 (7,633)	115,500 (19,024)	0.742
Superior quadrant	141,500 (7,503)	125,333 (12,754)	0.023^*^
Subfield 11	135,000 (28,921)	142,500 (27,406)	0.655
Subfield 12	143,667 (30,716)	128,833 (20,124)	0.346
Subfield 1	146,500 (22,801)	105,167 (8,841)	0.002^*^
Nasal quadrant	106,333 (28,444)	100,833 (27,140)	0.739
Subfield 2	118,167 (31,600)	104,667 (27,580)	0.449
Subfield 3	90,000 (22,821)	88,500 (26,614)	0.919
Subfield 4	109,667 (31,085)	109,833 (33,891)	0.993
Inferior quadrant	120,667 (14,137)	140,167 (24,975)	0.127
Subfield 5	109,333 (28,338)	138,000 (36,546)	0.160
Subfield 6	124,500 (24,023)	144,333 (38,609)	0.310
Subfield 7	127,667 (18,705)	138,667 (29,371)	0.457
Temporal quadrant	80,000 (28,213)	95,333 (29,351)	0.378
Subfield 8	86,000 (39,115)	92,667 (24,328)	0.730
Subfield 9	68,333 (21,267)	91,833 (44,256)	0.069
Subfield 10	86,167 (27,817)	102,000 (24,380)	0.319

RNFL: retinal nerve fiber layer thickness in *μ*.

1–12: three subfields for each quadrant numbered clockwise.

Group B: CMI patient's with syringomyelia.

Group D: healthy subjects.

^*^ *P* < 0.05, *t*-test.

**Table 4:** CMI patients with syringomyelia (Group B) versus healthy subject (Group D) OCT parameters, LE.

Variables	Average (*µ*)	Average (*µ*)	*P* value
	Standard deviation Group A	Standard deviation Group C	
RNFL	107,667 (8,287)	108,000 (10,770)	0.953
Superior quadrant	116,333 (12,972)	128,167 (13,152)	0.148
Subfield 11	138,500 (24,263)	132,333 (21,172)	0.649
Subfield 12	111,167 (21,830)	122,500 (18,641)	0.356
Subfield 1	99,333 (19,906)	129,667 (14,501)	0.013^*^
Temporal quadrant	68,667 (16,549)	88,500 (13,664)	0.047^*^
Subfield 2	76,333 (27,478)	95,000 (15,192)	0.176
Subfield 3	58,167 (17,116)	76,667 (23,441)	0.150
Subfield 4	71,000 (8,246)	93,833 (15,433)	0.010^*^
Inferior quadrant	134,500 (14,139)	128,667 (19,242)	0.563
Subfield 5	142,833 (26,828)	148,333 (28,794)	0.739
Subfield 6	148,500 (11,777)	134,667 (31,747)	0.341
Subfield 7	112,333 (24,712)	103,167 (18,946)	0.487
Nasal quadrant	106,833 (29,668)	86,833 (12,287)	0.060
Subfield 8	103,167 (28,435)	81,833 (15,562)	0.138
Subfield 9	100,500 (32,042)	76,500 (8,361)	0.030^**^
Subfield 10	116,500 (34,309)	102,333 (15,744)	0.700

RNFL: retinal nerve fiber layer thickness in *μ*.

1–12: three subfields for each quadrant numbered clockwise.

Group B: CMI patient's with syringomyelia.

Group D: healthy subjects.

^*^ *P* < 0.05, *t*-test.

^**^ *P* < 0.05, Mann-Whitney *U* test.
